# Implementation and first evaluation of PCIT parent-child-interaction-training in zurich

**DOI:** 10.1192/j.eurpsy.2021.613

**Published:** 2021-08-13

**Authors:** M. Zulauf Logoz, V. Mailänder

**Affiliations:** 1 Child And Adolescent Psychiatry, University of Zurich, Zürich, Switzerland; 2 Department Of Child And Adolescent Psychiatry And Psychotherapy, University of Zurich, Zürich, Switzerland

**Keywords:** Children, oppositional-defiant disorder, preschoolers, parent-child-interaction-training

## Abstract

**Introduction:**

Parent training is an evidence based and highly effective intervention for conduct disorders in children. Traditionally, only the parents participate in behavioral trainings, implementing the new skills in their homes on their own between the appointments. In some cases, this turns out as not intense enough.

**Objectives:**

Therefore, we recently implemented the German version of the PCIT Parent Child Interaction Training in our clinic in Zurich, Switzerland.

**Methods:**

PCIT is an evidence-based and highly effective intervention for children aged 2-7 years with conduct disorders (Zisser & Eyberg, 2010; Briegel, 2016). Parents visit the clinic weekly with their child and are directly supported in their interaction by the therapists. A special treatment room was set up for this intervention.

**Results:**

We will present our first experiences with this approach in the highly international and urban population of Zurich.

**Conclusions:**

Parents appreciate to work with their child while being directly coached by the therapists. Almost all parents achieved considerable progress in their skills and the conduct problems reduced over time.

